# Localization and regulation of yeast aldehyde dehydrogenase Ald4p structures

**DOI:** 10.1016/j.heliyon.2024.e39048

**Published:** 2024-10-09

**Authors:** Channarong Nasalingkhan, Naraporn Sirinonthanawech, Brian K. Sato, James E. Wilhelm, Chalongrat Noree

**Affiliations:** a Institute of Molecular Biosciences, Mahidol University 25/25 Phuttamonthon 4 Road, Salaya, Phuttamonthon, Nakhon Pathom, 73170, Thailand; b Department of Molecular Biology and Biochemistry, Charlie Dunlop School of Biological Sciences, University of California, Irvine, 2238 McGaugh Hall, Irvine, CA, 92697, USA; c Section of Cell and Developmental Biology, Division of Biological Sciences, University of California, San Diego, 9500 Gilman Drive (MC0347), La Jolla, CA, 92093-0347, USA

**Keywords:** Aldehyde dehydrogenase, Ald4p, Enzyme assembly, Yeast

## Abstract

Previously, we identified yeast strains, namely SWORD, showing more robust Ald4p-GFP filament formation than the typical *ALD4::GFP* strains. Here, we report that Ald4p-GFP in SWORD strains favorably polymerize into gigantic structures in the cytoplasm, despite the enzyme being established as a mitochondrial resident. In addition, we have found that nocodazole, a microtubule destabilizer, has no effect on Ald4p high-order assembly, suggesting no direct association between microtubule dynamics and Ald4p structure formation. Ald4p assembly cannot be induced by sodium azide treatment, indicating that ATP is not a primary effector of Ald4p polymerization. Interestingly, addition of exogenous acetaldehyde, a substrate of the enzyme, can significantly enhance the structure formation of Ald4p, implying that structure formation may be related to enzymatic activity.

## Graphical abstract

**Figure:**
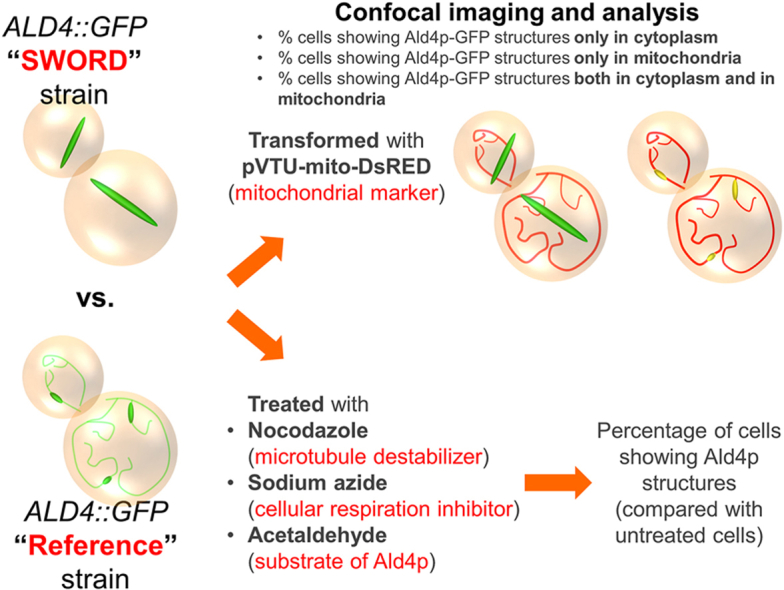

## 1 Introduction

Aldehyde dehydrogenases are a group of enzymes which can be utilized by living cells to catalyze the conversion of aldehydes into carboxylic acids. For example, highly toxic acetaldehyde can be oxidized by an aldehyde dehydrogenase to become a metabolically utilizable product, acetate. These enzymes help to prevent oxidative stress, cell damage and cell death, which can be triggered by the elevation of intracellular levels of aldehydes [1]. In humans, there are several genes responsible for the production of aldehyde dehydrogenases [2]. Human aldehyde dehydrogenases (ALDHs) are categorized, based on intracellular distribution, similarity of sequence and structure, and kinetic profile, into 3 main groups (cytosolic ALDH, mitochondrial ALDH, and microsomal ALDH) [3]. ALDH mutations can lead to several diseases, including alcoholic liver disease and some types of cancers [4,5]. In *Saccharomyces cerevisiae*, there are 5 genes (*ALD2*, *ALD3*, *ALD4*, *ALD5*, and *ALD6*) identified to encode yeast aldehyde dehydrogenases. Ald2p, Ald3p and Ald6p are classified as cytosolic enzymes, and Ald4p and Ald5p are known as mitochondrial enzymes. Each yeast aldehyde dehydrogenase responds to different regulatory and environmental conditions. The expression of cytosolic Ald2p and Ald3p can be stimulated by cellular stress or the presence of ethanol, and they function in alcohol oxidation and alanine biosynthesis [6,7]. Ald5p, a minor isoform of mitochondrial aldehyde dehydrogenases, is constitutively expressed and responsible for the regulation and biosynthesis of electron transport chain complexes [8]. The cytosolic Ald6p and the mitochondrial Ald4p (a major isoenzyme of mitochondrial aldehyde dehydrogenases) play a role in the oxidation of acetaldehyde into acetate [9].

**Fig. 1:**
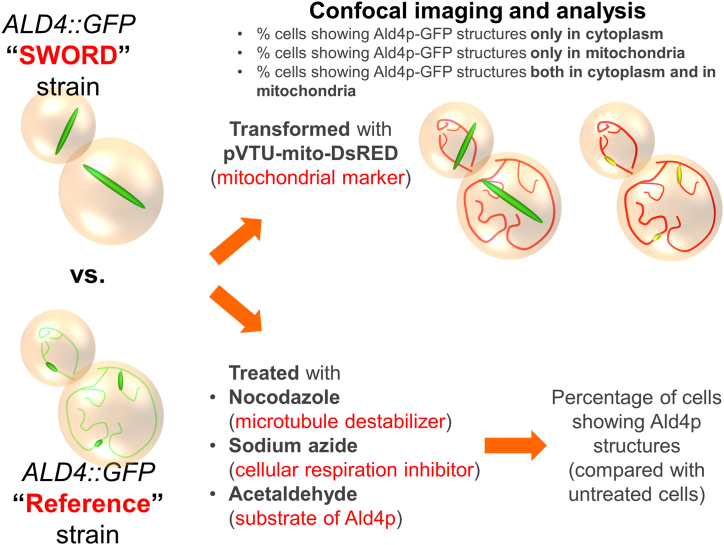
**Experimental Design.** In the first part, we use confocal microscopy to investigate the distribution or intracellular location of Ald4p-GFP assembled in the yeast *ALD4::GFP* ‘SWORD’ strains, relative to the yeast *ALD4::GFP* ‘reference’ strain. In the second part, we study if the Ald4p-GFP assembly could be regulated by nocodazole, sodium azide, and acetaldehyde treatments.

The mitochondrial aldehyde dehydrogenase Ald4p has been reported to assemble into visible structures in the yeast cells [10]. A version of the protein without its mitochondrial targeting sequence (MTS) can form very long filaments in the yeast cytoplasm, and the polymerization and depolymerization of these structures can be dynamically controlled by the availability of nutrients [11]. Our prior work with Ald4p has revealed that the activity-disrupted Ald4p is unable to polymerize into long filaments, suggesting that the filaments are made with active Ald4p molecules [12]. This same study generated wild type *ALD4* strains that exhibited a phenotype with more robust assembly of Ald4p-GFP filaments. These newly identified yeast *ALD4::GFP* strains, designated ‘SWORD’, were subjected to short-read whole genome sequencing to look for any genetic factor that might be associated with the SWORD phenotype. We found that alterations around the *FLO9* coding sequence could make the Ald4p-GFP structures longer than usual [13]. However, these SWORD clones of yeast *ALD4::GFP* have not been characterized for their cellular distribution and responses to environmental changes yet. In this study, we aim to investigate the distribution or intracellular location of Ald4p-GFP assembled in the yeast *ALD4::GFP* ‘SWORD’ strains, relative to the yeast *ALD4::GFP* ‘reference’ strain. This includes examining the regulation of Ald4p assembly leveraging nocodazole, sodium azide, and acetaldehyde treatments (Fig. 1).

## 2 Materials and methods

### 2.1 Bacterial and yeast strains

*Escherichia coli* One Shot™ MAX Efficiency™ DH5α-T1R competent cells (Thermo Fischer Scientific, USA) were used for propagation and maintenance of pVTU-mito-DsRED (a gift from J. Wilhelm, UC San Diego). The bacterial transformants were maintained in LB broth and/or on LB agar [1 % (w/v) Bacto™ tryptone (BD Biosciences), 0.5 % (w/v) Bacto™ yeast extract (BD Biosciences), 1 % (w/v) NaCl (Merck), and 1.5 % (w/v) Bacto™ agar (BD Biosciences)], supplemented with ampicillin (100 μg/mL) (PanReac AppliChem) at 37 °C. pVTU-mito-DsRED was then extracted using Presto™ Mini Plasmid Kit (Geneaid), following the manufacturer's instructions.

Yeast *ALD4::GFP; kanR* ‘reference’ was constructed as previously described [11], and yeast *ALD4::GFP; kanR* ‘SWORD’ was isolated during the construction of yeast strains expressing mutant Ald4p-GFP [12]. Yeast cultures were maintained in YPD broth and/or on YPD agar [(2 % (w/v) Bacto™ peptone (BD Biosciences), 1 % (w/v) Bacto™ yeast extract, 2 % (w/v) glucose (Sigma-Aldrich), and 1.5 % (w/v) Bacto™ agar], supplemented G418 (400 μg/mL) (PanReac AppliChem) at 30 °C.

### 2.2 Transformation of yeast *ALD4:GFP* ‘reference’ and ‘SWORD’ with pVTU-mito-DsRED

To label the yeast mitochondria, pVTU-mito-DsRED was introduced into the two yeast strains, *ALD4::GFP* ‘reference’ and ‘SWORD’. The overnight yeast cultures were prepared in 3 mL YPD broth and grown at 30 °C with shaking. One mL of each overnight culture was then transferred into a culture flask having 30 mL fresh YPD broth. The flasks were incubated at 30 °C with shaking for 4 h to grow the cells to the mid log phase (OD_600_ ∼ 0.4–0.6). The fresh competent yeast cells were subsequently prepared using the protocol previously described in Ref. [13]. Five hundred ng of pVTU-mito-DsRED was used for each transformation. The yeast transformants carrying pVTU-mito-DsRED were selected on the agar made with uracil-dropout synthetic medium ‘SC-uracil’ (Sigma-Aldrich) and 2 % (w/v) glucose.

### 2.3 Yeast cell imaging

To determine the localization and assembly of Ald4p-GFP in yeast *ALD4::GFP* ‘reference’ and ‘SWORD’ strains, the cells were directly scraped from their agar plates and resuspended in an appropriate volume of 1xPBS (Merck). The preparation of yeast samples on the microscope slides was previously described in Ref. [13]. Images were taken with the Carl Zeiss LSM800 using Plan-Apochromat 63x/1.4 Oil DIC ∞/0.17 objective lens with Zen Blue software version 2.1.

### 2.4 Nocodazole, sodium azide, and acetaldehyde treatments

Yeast cultures (*ALD4::GFP* ‘reference’ and ‘SWORD’) were grown to mid log phase at 30 °C in YPD broth. One mL of each was transferred into a sterile microcentrifuge tube, and 10 μL of 5 mg/mL nocodazole solution (Sigma-Aldrich) was added to the tube. For the control, 10 μL of DMSO (Sigma-Aldrich) was added. The treated cells were incubated at room temperature with shaking for 30 min. The cells were then fixed by adding 100 μL of 37 % formaldehyde solution to each tube. After 15-min incubation with shaking at room temperature in the dark, the cells were briefly washed in 1 mL sterile water twice (centrifugation was used to pellet the cells at 3381 g for 2 min). After the last wash, the cells were resuspended in 1xPBS. Their wet slides were prepared to count the number of cells showing Ald4p-GFP assembly under the fluorescence microscope (DeltaVision Ultra with Olympus PlanApo N 60X/1.42 Oil objective lens). The data (number of cells showing Ald4p-GFP structures/total number of cells counted) were reported as average ± SEM, calculated from 3 independent experiments (in each experiment, at least 250 cells were inspected for each treatment condition).

For the sodium azide treatment, 10 μL of 1M sodium azide (Sigma-Aldrich) was used to treat the log-phase cells (1 mL) for 15 min, whereas 10 μL of sterile water was used as a control. For the acetaldehyde treatment, 5 μL of acetaldehyde (Sigma-Aldrich, 402788, ≥99.5 %, density 0.785 g/mL at 25 °C) was used to treat the log-phase cells (1 mL) for 15 min, and 5 μL of sterile water was used as a control.

Paired *t*-test [GraphPad Prism version 10.1.0 (316)] was used to check statistical significance between treated and control samples.

## 3 Results and discussion

### 3.1 Yeast *ALD4:GFP* ‘SWORD’ strains form Ald4p-GFP structures in the cytoplasm more ubiquitously than yeast *ALD4:GFP* ‘reference’ strains

In previous work, we reported the identification of yeast *ALD4::GFP* strains, namely ‘SWORD’, showing more robust assembly of Ald4p-GFP than the typical *ALD4::GFP* strains [13]. The Ald4p-GFP in the ‘SWORD’ strains can assemble into very long filaments (almost from one side to the other side of the cells), an intriguing finding as the enzyme is known to be a mitochondrial resident [14,15].

**Fig. 2:**
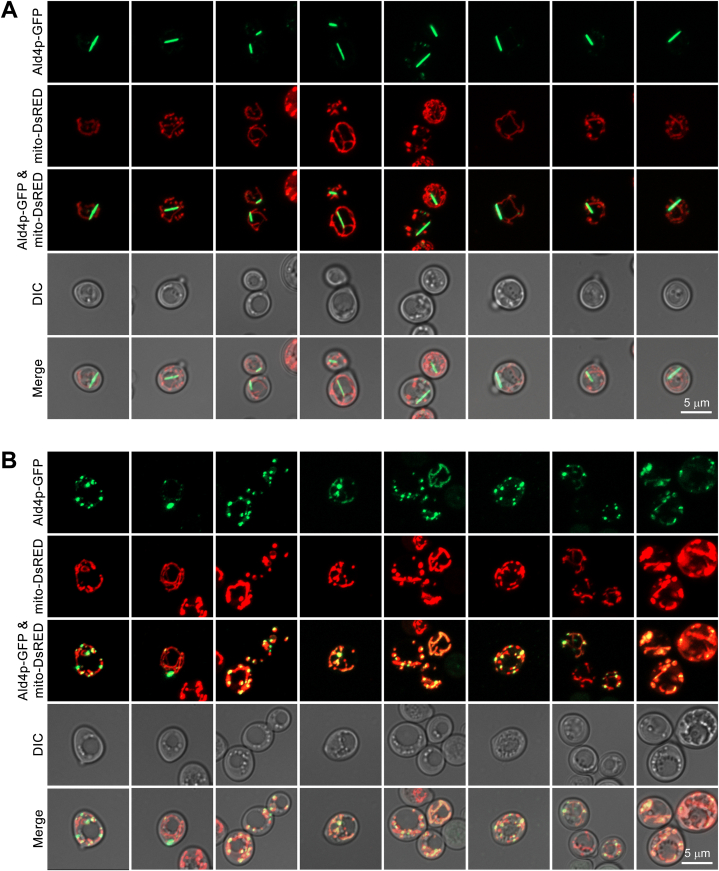
**Ald4p-GFP structures are abundantly found in the cytoplasm of yeast *ALD4::GFP* (SWORD) strains.** Yeast *ALD4::GFP* ‘SWORD’ (**A**) and *ALD4::GFP* ‘reference’ (**B**) strains were transformed with pVTU-mito-DsRED (used for labelling the mitochondria). Live cells were directly scraped from the selective agar plates, resuspended in 1xPBS, and imaged with Zeiss LSM800. The Z-stack images of each field of view were compressed into a 2D image using Zen (blue) software. These are representative images of the data summarized in Table 1.

To first determine whether the Ald4-pGFP structures are mitochondrially-localized, we introduced pVTU-mito-DsRED (a DsRED mitochondria label) to the ‘SWORD’ and ‘reference’ strains (Fig. 2A–B). Surprisingly, we found that Ald4p-GFP structures were outside of the mitochondria in both ‘SWORD’ and ‘reference’ strains (Fig. 2B). Within the structure-forming population of ‘reference’ cells, 53 % of cells possessed Ald4p-GFP structures formed in both the mitochondria and cytoplasm, 20 % of cells possessed of Ald4p-GFP structures localized to the mitochondria only, and 27 % of cells had Ald4p-GFP structures found in the cytoplasm only (Table 1).

**Table 1:** Distribution of Ald4p-GFP structures in the *ALD4::GFP* yeast strains (SWORD and reference) (transformed with pVTU-mito-dsRED).

Yeast strain	Number of cells with				
	Red fluorescent signal expressed in the mitochondria (pVTU-mito-dsRED)	Ald4p-GFP structures (regardless of intracellular location)	Ald4p-GFP structures in the cytoplasm only	Ald4p-GFP structures in the mitochondria only	Ald4p-GFP structures in both cytoplasm and mitochondria
*ALD4::GFP* (**SWORD**)	**324**	**280** (280/324 or **86.4 %**^a^)	**160** (160/280 or **57.1 %**^b^)	**55** (55/280 or **19.6 %**^b^)	**65** (65/280 or **23.2 %**^b^)
*ALD4::GFP* (**Reference**)	**208**	**156** (156/208 or **75.0 %**^a^)	**42** (42/156 or **26.9 %**^b^)	**31** (31/156 or **19.9 %**^b^)	**83** (83/156 or **53.2 %**^b^)

**Note:** This table shows cumulative data gathered from 2 different clones (of each yeast strain) and 2 independent experiments.

a indicates % cells with Ald4p-GFP structures (relative to the total number of cells positively showing red fluorescent signal in the mitochondria).

b indicates the proportion of cells having Ald4p-GFP structures in the indicated intracellular compartment (relative to the total number of cells having Ald4p-GFP structures regardless of intracellular location).

**Fig. 3:**
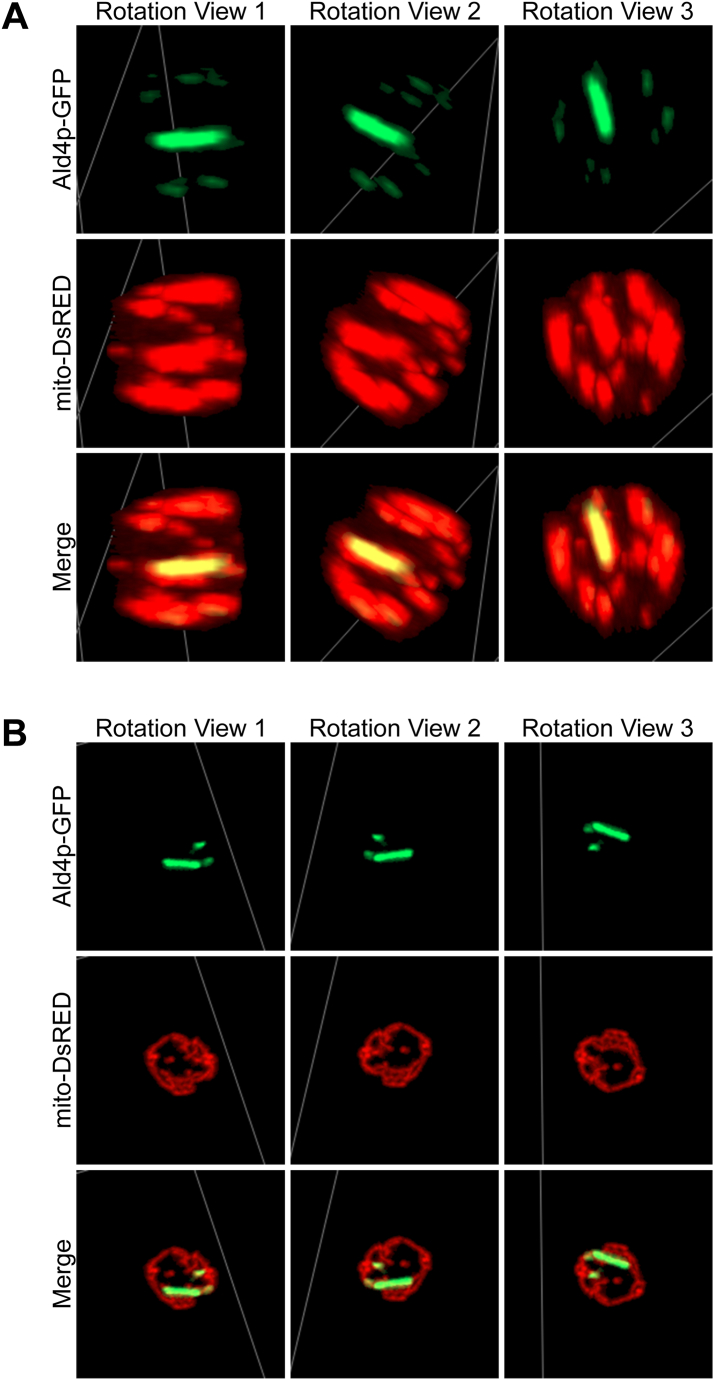
**Three dimensional images of yeast *ALD4::GFP* ‘SWORD’ strains carrying pVTU-mito-DsRED confirm the capability of Ald4p-GFP to form structures in both cytoplasm and mitochondria.** The Z-stack images of live yeast cells (*ALD4::GFP* ‘SWORD’ strains) were rotated in 3 different angles of view to show that Ald4p-GFP can assemble in the mitochondria (**A**) and in the cytoplasm (**B**). These images were the representatives of the data summarized in Table 1. Note: Scale bars cannot be drawn as these images were snapshots from 3D rotations (cells are not in the same depth when being rotated in different angles) by the 3D viewing function (Zen Blue).

Interestingly, the fraction of cells that possessed Ald4p-GFP structures only in the cytoplasm was 2.1-fold higher in the ‘SWORD’ strain relative to the ‘reference’ clones (57.1 % vs. 27.9 %). When looking at the number of cells in the sample with Ald4p-GFP structures in the mitochondria only, they were similar between strains (Table 1) (representative images of Ald4p-GFP filaments formed within the mitochondria and in the cytoplasm displayed in Fig. 3A and B, respectively). The number of cells showing the assembly of Ald4p-GFP structures in both the cytoplasm and mitochondria were 2.3-fold lower in the ‘SWORD’ than the ‘reference’ *ALD4::GFP* strains (23.2 % vs 53.2 %) (Table 1). Based on these observations, we can conclude that, in addition to having significantly longer filaments [13], Ald4p-GFP is more likely to polymerize in the cytoplasm in ‘SWORD’ versus ‘reference’ *ALD4::GFP* strains.

Aldehyde dehydrogenases are not only utilized for the conversion of aldehyde substrates into carboxylic acids, but they can also help maintain the NAD+/NADH ratio inside the cells [14]. It is possible that right after being synthesized by ribosomes in the cytoplasm, the newly synthesized Ald4p molecules are experiencing cytoplasmic microenvironments where the activity of Ald4p is required. For example, if the concentration of intracellular aldehydes or the NAD+/NADH ratio is out of normal physiological range, assembly of Ald4p could help stimulate its biochemical function.

### 3.2 The assembly of Ald4p-GFP can be altered by addition of exogenous acetaldehyde, but not by nocodazole and sodium azide treatments

**Fig. 4:**
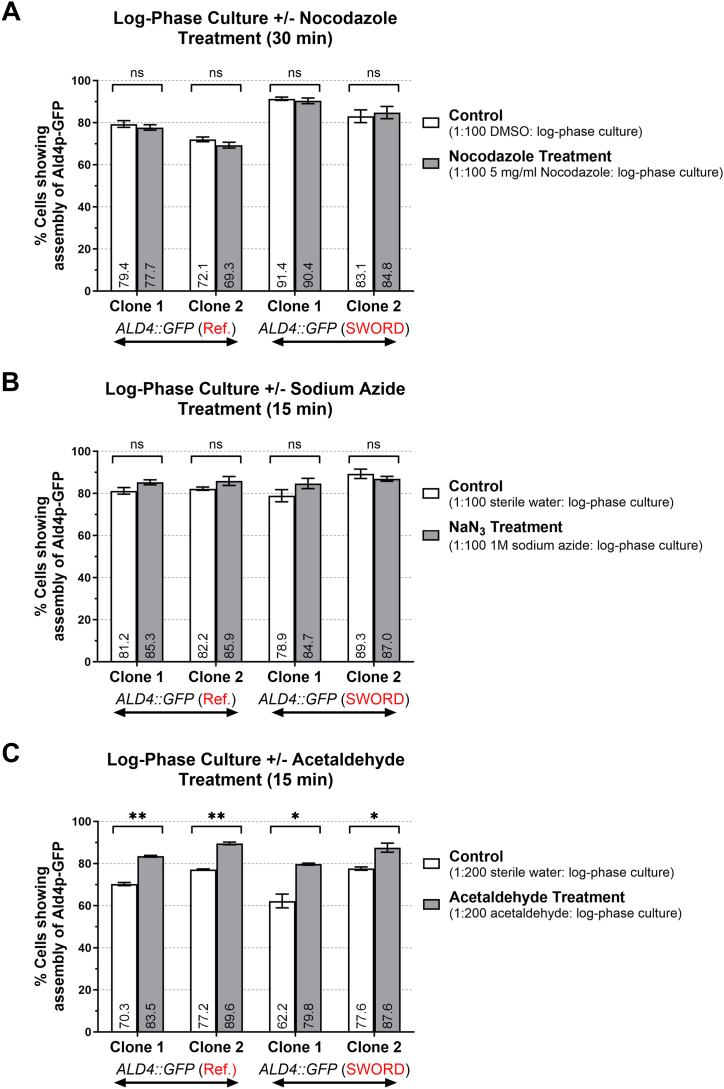
**Exogenous acetaldehyde can trigger the assembly of Ald4p-GFP, whereas destabilization of microtubules and depletion of ATP levels have no effect on Ald4p-GFP assembly.** Log-phase *ALD4::GFP* ‘reference’ and ‘SWORD’ cultures were prepared and then treated with nocodazole (30 min) to destabilize microtubules (**A**), sodium azide (15 min) to deplete intracellular ATP levels (**B**), or acetaldehyde, known as one of Ald4p substrates (15 min) (**C**). The cells were subsequently fixed with formaldehyde (3.36 % final concentration) before counting the number of cells having Ald4p-GFP structures. Three independent experiments were performed. Data were plotted and statistical analyses were performed using paired *t*-test (GraphPad Prism). ∗ indicates statistically significant difference between two groups when p-value ≤0.05, ∗∗ when p-value ≤0.01.

As metabolic enzymes have been found to be associated with structural or cytoskeletal proteins [[16], [17], [18], [19]], we next asked if the yeast Ald4p did so as well. We treated log-phase ‘SWORD’ and ‘reference’ *ALD4::GFP* strains with 50 μg/mL nocodazole for 30 min to disrupt the microtubule formation [20]. Nocodazole treatment had no effect on the assembly/disassembly of Ald4p in both ‘SWORD’ and ‘reference’ strains (Fig. 4A). Thus, we conclude that the Ald4p assembly does not depend on the availability and dynamics of microtubules in the cells.

Next, we tested whether intracellular ATP levels affected Ald4p structure formation by treating log-phase cells with 10 mM sodium azide (an inhibitor of ATP production). Both CTP synthetase and asparagine synthetase structure formation are sensitive to sodium azide treatment [21,22], although ATP is an effector in their enzymatic functions, whereas aldehyde dehydrogenases does not require ATP for catalytic activity. After 15-min incubation with sodium azide, similar numbers of cells in the treated and untreated conditions exhibited Ald4p-GFP structures in both ‘SWORD’ and ‘reference’ clones (Fig. 4B). Therefore, there does not appear to be a correlation between cellular energy levels and the Ald4p assembly.

Finally, we tested the effects of exogenous acetaldehyde on Ald4p structure assembly. This was previously tested with a mutant *ALD4* gene that lacked the mitochondrial targeting sequence (MTS). In log-phase cultured cells, the MTS-deleted Ald4p formed cytoplasmic structures, but only upon acetaldehyde addition (<2 % cells showing structures before treatment, >98 % after treatment) [12]. When log-phase cells expressing wild type Ald4p-GFP were treated with exogenous acetaldehyde, we observed a significant increase in high-order assembly in both ‘SWORD’ and ‘reference’ strains (19.7 % and 17.4 % increase, respectively) (Fig. 4C). This finding is consistent with our previous mutation study of Ald4p that disruption of the Ald4p activity could inhibit the assembly of the enzymes [12]. Altogether, we propose that Ald4p molecules assemble together in order to promote their enzymatic activity.

## 4 Conclusion

Ald4p is an enzyme previously found capable of forming supramolecular complexes with certain enzymes in the TCA cycle, including Cit1p (citrate synthase), Mdh1p (malate dehydrogenase), Fum1p (fumarase), and Sdh1p (flavoprotein subunit of succinate dehydrogenase) [14]. Here, we provide imaging data to demonstrate that Ald4p, despite a long-held belief that it is a mitochondrial enzyme, can actually assemble in the cytoplasm. In addition, we show that Ald4p assembly can be stimulated by one of its substrates, acetaldehyde. Thus, it points out the importance of the enzyme assembly which is tightly associated with the regulation of enzymatic activity.

While early views of the regulatory roles of filament formation in regulating enzyme activity were as a sequestering mechanism to keep the enzyme in an inactive state, more recent studies have broadened the potential roles for filament formation. For instance, human IMPDH2 assembles in an active conformation where filament formation makes the enzyme less responsive to feedback inhibition [23]. Our observation that substrates trigger cytoplasmic Ald4p filament formation suggests that the filament may be assembling in an active state. Interestingly, recent cryoEM studies have found that Ald4p forms both helical and non-helical filaments in mitochondria during meiosis [24]. These results raise the possibility that different filament assemblies might display different regulatory responses to substrates and products. These regulatory possibilities are increased by the fact that Ald4p forms filaments in the cytoplasm and mitochondria. Future studies using mutations that specifically block helical vs non-helical Ald4p filaments could help dissect the structural subtype of Ald4p filaments in the cytoplasm and help determine if aldehyde triggers both types of Ald4p filaments to assemble.

Ald4p is not unique in its ability to partition between the cytoplasm and mitochondria. Both human and yeast fumarase are known to partition into both the cytoplasm and mitochondria with distinct roles in each compartment. The partitioning of fumarase between mitochondria and the cytoplasm is determined by the rate of translocation [25]. Interestingly, previous studies predicting dual localized proteins did not identify Ald4p as being dual localized to the cytoplasm and mitochondria suggesting that it might partition via a different regulatory mechanism [26]. Our finding that the SWORD strain background regulates the amount of cytoplasmic Ald4p polymer suggests that the SWORD strain would be an excellent tool for dissecting the partitioning mechanism. This will be a particularly powerful approach given the diversity of potential mechanisms for generating dual localization: multiple targeting signals that are recognized by the sorting machinery of different organelles, and retrotranslocation. Future work combining mutations that disrupt filament formation with mapping of cytoplasmic and mitochondrial targeting sequences will help determine if filament formation plays a role in partitioning and create the necessary reagents to separate the role of Ald4p in mitochondria from its role in the cytoplasm.

## Funding

This research project was supported by 10.13039/501100004156Mahidol University (to C. Noree) and the Development and Promotion of Science and Technology Talents Project (DPST) (to C. Nasalingkhan and C. Noree).

## CRediT authorship contribution statement

**Channarong Nasalingkhan:** Visualization, Validation, Methodology, Investigation, Funding acquisition, Formal analysis. **Naraporn Sirinonthanawech:** Visualization. **Brian K. Sato:** Writing – review & editing, Writing – original draft. **James E. Wilhelm:** Writing – review & editing, Writing – original draft. **Chalongrat Noree:** Writing – review & editing, Writing – original draft, Visualization, Validation, Supervision, Resources, Project administration, Methodology, Investigation, Funding acquisition, Formal analysis, Conceptualization.

## Declaration of competing interest

The authors declare that they have no known competing financial interests or personal relationships that could have appeared to influence the work reported in this paper.

## Data Availability

All data generated or analyzed during this study are included in this published article and its supplementary information files.
