# Supplementary material for: Localization and regulation of yeast aldehyde dehydrogenase Ald4p structures
Source: Heliyon. 2024 Oct 9;10(20):e39048. doi: 10.1016/j.heliyon.2024.e39048 (PMC11620093; doi:10.1016/j.heliyon.2024.e39048)
Supplement: Multimedia component 1 [file mmc1.pdf]

Supplementary Information

Localization and regulation of yeast aldehyde dehydrogenase Ald4p structures

Channarong Nasalingkhan, Naraporn Sirinonthanawech, Brian K. Sato, James E. Wilhelm, Chalongrat Noree

Table S1. Numerical and statistical data of two yeast strains, *ALD4::GFP* (reference) and *ALD4::GFP* (SWORD), used for plotting graphs shown in **Fig. 4A-4C**.

Log-phase culture treated with nocodazole (30 min)					
**Yeast strain**	**Clone #**	**% Cells with Ald4p-GFP structures** (average ± SEM)		**P-value (two-tailed; paired t test)**	**Significantly different (< 0.05)?**
		**(1:100) DMSO: log-phase culture**	**(1:100) 5 mg/mL nocodazole: log-phase culture**		
ALD4::GFP (reference)	1	79.4 ± 1.58	77.7 ± 1.26	0.1583	No (ns)
	2	72.1 ± 1.12	69.3 ± 1.39	0.3828	No (ns)
ALD4::GFP (SWORD)	1	91.4 ± 0.81	90.4 ± 1.36	0.6746	No (ns)
	2	83.1 ± 3.05	84.8 ± 2.91	0.2253	No (ns)
**Log-phase culture treated with sodium azide (15 min)**					
**Yeast strain**	**Clone #**	**% Cells with Ald4p-GFP structures** (average ± SEM)		**P-value (two-tailed)**	**Significantly different (< 0.05)?**
		**(1:100) sterile water: log-phase culture**	**(1:100) 1M NaN_3: log-phase culture**		
ALD4::GFP (reference)	1	81.2 ± 1.57	85.3 ± 1.16	0.2110	No (ns)
	2	82.2 ± 0.78	85.9 ± 2.12	0.1246	No (ns)
ALD4::GFP (SWORD)	1	78.9 ± 2.87	84.7 ± 2.43	0.3286	No (ns)
	2	89.3 ± 2.23	87.0 ± 1.16	0.4456	No (ns)
**Log-phase culture treated with acetaldehyde (15 min)**					
**Yeast strain**	**Clone #**	**% Cells with Ald4p-GFP structures** (average ± SEM)		**P-value (two-tailed)**	**Significantly different (< 0.05)?**
		**(1:200) sterile water: log-phase culture**	**(1:200) acetaldehyde: log-phase culture**		
ALD4::GFP (reference)	1	70.3 ± 0.75	83.5 ± 0.39	0.0066	Yes (**)
	2	77.2 ± 0.23	89.6 ± 0.63	0.0011	Yes (**)
ALD4::GFP (SWORD)	1	62.2 ± 3.28	79.8 ± 0.47	0.0252	Yes (*)
	2	77.6 ± 0.81	87.6 ± 2.15	0.0284	Yes (*)

Note: ns (not significant, p-value > 0.05), * (p-value ≤ 0.05), ** (p-value ≤ 0.001).
